# Failure of thyroid hormone treatment to prevent inflammation-induced white matter injury in the immature brain

**DOI:** 10.1016/j.bbi.2013.11.005

**Published:** 2014-03

**Authors:** Anne-Laure Schang, Juliette Van Steenwinckel, Didier Chevenne, Marten Alkmark, Henrik Hagberg, Pierre Gressens, Bobbi Fleiss

**Affiliations:** a Inserm U676, 75019 Paris, France; b Université Paris Diderot, Faculté de Médecine, 75019 Paris, France; c PremUP, 75014 Paris, France; d Service de biochimie et hormonologie, Hôpital Robert Debré, 75019 Paris, France; e Department of Clinical Sciences, Sahlgrenska Academy/East Hospital, 416 85 Gothenburg, Sweden; f Department of Perinatal Imaging and Health, Division of Imaging Sciences and Biomedical Engineering, King’s College London, King’s Health Partners, St. Thomas’ Hospital, London SE1 7EH, United Kingdom

**Keywords:** Prematurity, Thyroxine, Oligodendrocyte, Myelination, Neuroprotection

## Abstract

Preterm birth is very strongly associated with maternal/foetal inflammation and leads to permanent neurological deficits. These deficits correlate with the severity of white matter injury, including maturational arrest of oligodendrocytes and hypomyelination. Preterm birth and exposure to inflammation causes hypothyroxinemia. As such, supplementation with thyroxine (T4) seems a good candidate therapy for reducing white matter damage in preterm infants as oligodendrocyte maturation and myelination is regulated by thyroid hormones.

We report on a model of preterm inflammation-induced white matter damage, in which induction of systemic inflammation by exposure from P1 to P5 to interleukin-1β (IL-1β) causes oligodendrocyte maturational arrest and hypomyelination. This model identified transient hypothyroidism and wide-ranging dysfunction in thyroid hormone signalling pathways. To test whether a clinically relevant dose of T4 could reduce inflammation-induced white matter damage we concurrently treated mice exposed to IL-1β from P1 to P5 with T4 (20 μg/kg/day). At P10, we isolated O4-positive pre-oligodendrocytes and gene expression analysis revealed that T4 treatment did not recover the IL-1β-induced blockade of oligodendrocyte maturation. Moreover, at P10 and P30 immunohistochemistry for markers of oligodendrocyte lineage (NG2, PDGFRα and APC) and myelin (MBP) similarly indicated that T4 treatment did not recover IL-1β-induced deficits in the white matter.

In summary, in this model of preterm inflammation-induced white matter injury, a clinical dose of T4 had no therapeutic efficacy. We suggest that additional pre-clinical trials with T4 covering the breadth and scope of causes and outcomes of perinatal brain injury are required before we can correctly evaluate clinical trials data and understand the potential for thyroid hormone as a widely implementable clinical therapy.

## Highlight

• Thyroid hormone treatment did not recover deficits in oligodendrocyte maturation and myelination in a mouse model of preterm inflammation-induced white matter damage.

## 1 Introduction

Premature birth often leads to lifelong sensory and motor deficits, cognitive and learning impairments and behavioural disturbances (Arnaud et al., 2007). These neurological impairments are typically associated with damage to the white matter, including a maturational blockade (but not loss) of oligodendrocytes (Billiards et al., 2008; Buser et al., 2012; Verney et al., 2012) and smaller volumes on MRI (Counsell et al., 2003; Anjari et al., 2007).

Among the various factors considered to contribute to white matter injury in preterm infants and to represent a viable therapeutic strategy, augmentation of thyroid hormones levels (TH) is particularly credible approach for several reasons. These include that up to 85% of preterm infants suffer from hypothyroidism that can persist for several weeks after birth (Reuss et al., 1996; Leviton et al., 1999; Simpson et al., 2005). This transient hypothyroidism of prematurity is associated with increased risks for white matter damage and poor long-term neurological outcome. Hypothyroidism is a consequence of prematurity (Radetti et al., 2004), and is induced by inflammation in both adults and neonates (De Felice et al., 2005). Compounding the vulnerability of the preterm population, inflammation due to maternal foetal/inflammation (such as chorioamnionitis) is a leading cause premature labour (Hillier et al., 1993; Dammann and Leviton, 2004; Goldenberg et al., 2008; Wu et al., 2009).

THs are essential for brain development due to their prolific regulation of gene expression, and even a short period of deficiency during development can lead to irreversible brain damage (Berbel et al., 2010). Supporting the idea that supplementation of TH may be a viable neurotherapeutic strategy, T4 is a potent inducer of oligodendrocyte maturation and (re)myelinationin in animal models of demyelination and *in vitro* (Gravel and Hawkes, 1990; Baas et al., 1997; Jones et al., 2003; Lin et al., 2011; Dugas et al., 2012).

Three randomized clinical trials have been undertaken to test the efficacy of thyroxin (T4) supplementation in reducing neurological deficits in preterm infants (Chowdhry et al., 1984; Vanhole et al., 1997; van Wassenaer et al., 2005). The two small trials (<40 infants) demonstrated no effect of T4 treatment, but the largest and most recent revealed subtle improvements in learning out to ten years of age, but only for infants born below 27 weeks gestational age (van Wassenaer et al., 2005). A new randomized clinical trial powered to test this potential gestational age-dependent effect began in 2012 (Ng et al., 2013). However, due to uncertainty regarding the population of infants likely to benefit from T4 treatment and what mechanism underpins this variation, more research is required in experimental models to understand the situations in which T4 treatment may have the greatest therapeutic efficacy. TH therapy has been trialled in experimental models of perinatal excitotoxicity and hypoxia-ischemia (HI) (Sarkozy et al., 2007; Hung et al., 2013). However, the efficacy of TH has not been tested in a model of preterm injury induced by inflammation alone, and results obtained in this context would improve our interpretation of clinical trial data.

As such, we have utilized a model of preterm inflammation-induced white matter injury (mice treated with interleukin-1β [IL-1β] from postnatal day [P] 1 to 5). These animals display a long-lasting myelination deficit linked to a blockade of oligodendrocyte differentiation, accompanied by cognitive defects and MRI abnormalities (Favrais et al., 2011). In this study we demonstrate that this model recapitulates the clinically observed transient hypothyroidism observed in preterm infants and induces wide-ranging dysregulation of expression for TH signalling and responsive genes. Nevertheless, treatment with a clinically relevant dose of T4 is unable to recover the inflammation-induced white matter deficits.

## 2 Materials and methods

### 2.1 Animals and drug administration

Experimental protocols were approved by the institutional guidelines of the Institut National de la Santé et de la Recherche Scientifique (Inserm) France, and met the guidelines for the United States Public Health Service’s Policy on Humane Care and Use of Laboratory Animals (NIH, Bethesda, Maryland, USA). Experiments were performed using OF1 strain mice purchased from Charles River (L’Arbresle, France) and born in our animal facility. Animals were housed under a 12 h light-dark cycle, had access to food and water *ad libitum* and were weaned into same sex groups at P21. On P1 pups were sexed and where necessary litters were culled to 9–11 pups. Assessments of injury and outcomes were made only in male animals and all pups within a litter received identical treatment to reduce any effects of differing maternal care. IL-1β exposure was carried out as previously described (Favrais et al., 2011). Briefly, mice received twice a day from P1 to P4 and once on P5 a 5 μl intra-peritoneal injection of 10 μg/kg/injection recombinant mouse IL-1β in phosphate buffered saline (PBS; R&D Systems, Minneapolis, MN) or PBS alone. For TH trials, each morning only from P1–P5 pups were co-injected with IL-1β and 20 μg/kg/injection of T4 (T0397, Sigma-Aldrich) or at each of the 9 treatments with IL-1β and 20 μg/kg/injection of Triiodthyronine (T3; T6397, Sigma–Aldrich, Lyon, France). The dosages of T4 and T3 are similar to those used clinically (Vanhole et al., 1997; La Gamma et al., 2009), and approximate the euthyroid dose for mice based on previous serum measurements (Hoath et al., 1983; Mori et al., 2006; Rodrigues et al., 2013) and a half life of T4 of 13–16 h in the neonatal mouse (van Buul-Offers et al., 1983).

### 2.2 Measurements of free-T3 and free-T4 in serum

At P5 and P10, pups were decapitated and blood samples were collected in non-heparinized tubes. Blood samples were allowed to coagulate for 10 min at room temperature and were centrifuged for 7 min at 4000 rpm. Sera were collected and stored at −20 °C before free-T3 and free-T4 levels were measured using Siemens Advia Centaur CP immunochemiluminescent assays (Siemens Healthcare Diagnostics SAS, Saint-Denis, France).

### 2.3 Neural tissue dissociation and O4+ magnetic-activated cell sorting

At P5 and P10, brains were collected for cell dissociation and O4-positive cell enrichment using a magnetic coupled antibody extraction technique (MACS), as previously described and according to the manufacturer’s protocol (Jungblut et al., 2012) (Miltenyi Biotec, Bergisch Gladbach, Germany). The O4 antigen is expressed on the cell surface of pre-oligodendrocytes (Back et al., 2001). In brief, after removing the cerebellum and olfactory bulbs the brains were pooled (*n* = 3 at P5 and *n* = 2 at P10) and dissociated using the Neural Tissue Dissociation Kit containing papain. From the resulting brain homogenate O4-positive cells were enriched by MACS, using the anti-O4 MicroBeads and after elution the isolated cells were centrifuged for 5 min at 600*g* and conserved at −80 °C. The purity of the eluted O4-positive fraction was verified using qRT-PCR for glial fibrillary acid protein (GFAP), neuronal nuclear antigen (NeuN) and ionizing calcium binding adapter protein (Iba1) and revealed gene expression levels 95% lower than found in the respective primary cultures of astrocytes, neurons or microglia.

### 2.4 Microarray analysis and quantitative reverse-transcriptase polymerase-chain reaction

Microarray analysis, including RNA extraction and quality assurance, was performed by Miltenyi Biotec on a total of 24 MACS extracted O4 enriched cell samples from P5 or P10 mice exposed to IL-1β or PBS. Preparation of samples for array analysis and quantitative reverse-transcriptase polymerase-chain reaction (qRT-PCR), primer design, and PCR protocol, were similar to that previously described (Husson et al., 2005; Chhor et al., 2013). Primer sequences are given in Suppl. Table 1. *Gapdh* (glyceraldehyde-3-phosphate dehydrogenase gene) was chosen to standardize the quantitative experiments based on reference gene suitability testing. The relative quantities are expressed as the specific ratio between the gene of interest and *Gapdh*.

### 2.5 Immunohistochemistry and immunofluorescence

At P10, brains were collected for preparation of frozen sections following intracardial perfusion with 4% paraformaldehyde-phosphate buffer solution under isofluorane anaesthesia. Brains were post-fixed for 4 h at room temperature and then following at least three days in 30% sucrose in PBS the brains were embedded in 15% sucrose-7.5% gelatine solution and frozen at −80 °C before sectioning at 16 μm. At P30 brains were processed to paraffin sections by immediate immersion for 6-7 days in 4% formaldehyde at room temperature before dehydration, embedding in paraffin and sectioning at 12 μm. Primary antibodies used were anti-Myelin Basic Protein (MBP, 1:500, Chemicon, Temecula, CA, USA), anti-Platelet Derived Growth Factor Receptor-alpha (PDGFRα, 1:500, BD Biosciences, San Jose, CA, USA), anti-Adenomatosis Polyposis Coli (APC, 1:2000, Calbiochem, CA, USA) and anti-NG2 (1:200, Chemicon). Immunohistochemistry and Immunofluorescence staining were performed as previously described (Favrais et al., 2011). Nuclei were counterstained for immunofluorescence with DAPI (Sigma–Aldrich). All analyses were performed by an experimenter blind to treatment group. The intensity of MBP immunostaining was assessed using densitometric analysis as previously described (Favrais et al., 2011). Cell counts for NG2, PDGFRα and APC were performed in four sections per animal for each defined brain structure and are expressed as the percentage of positive cells per total number of nuclei.

### 2.6 Statistical and microarray analysis

Quantitative data are expressed as mean ± SEM values for each treatment group and group numbers are indicated within the text or legends. Comparisons of results were conducted by using nonparametric Mann–Whitney test (Prism 4.01; Graphpad Software, San Diego, CA). The Agilent feature extraction software was used to process microarray image files. Only signal intensities above background were included. Signal intensity values were background subtracted and uploaded following instructions by Miltenyi Biotec GmbH (Stefan Tomiuk) and Perkin Elmer (Matt Hudson) into GeneSifter Analysis Edition v4.0 (http://login.genesifter.net/) for further analysis as previously described (Gustavsson et al., 2007). The pre-processed signal intensity values were median normalized and the gene expression in IL-1β and PBS controls were compared at P5 and P10 using *t*-test (*p* < 0.05) with Benjamini–Hochberg multiple testing correction.

## 3 Results

### 3.1 Perinatal systemic inflammation induces a transient hypothyroidism and dysregulates thyroid signalling pathways

**Fig. 1:**
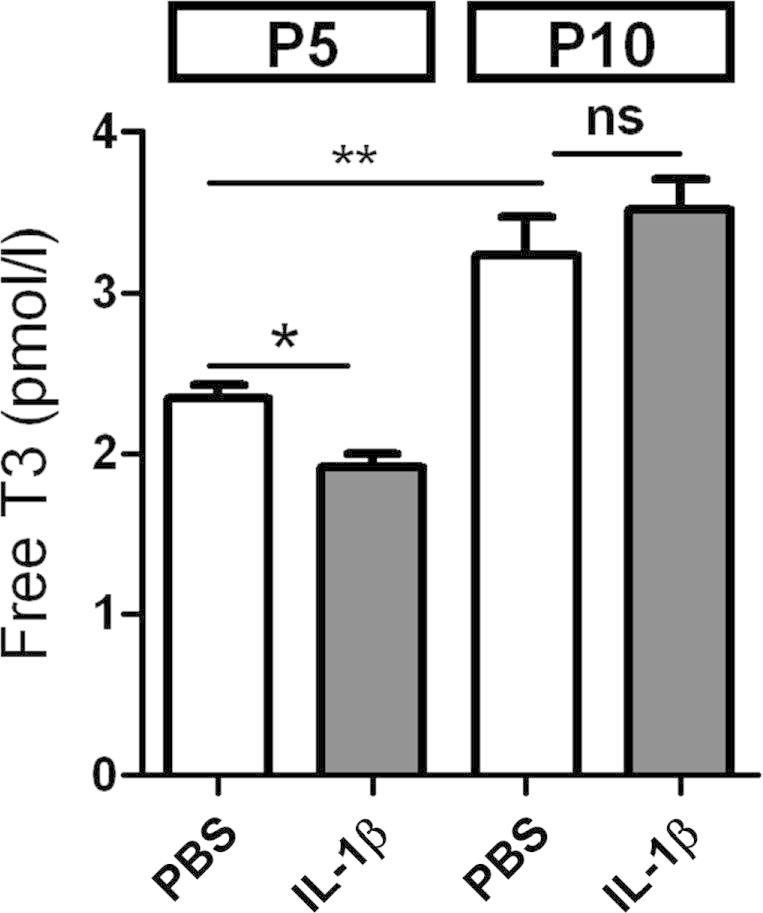
Serum levels of free-T3 were transiently decreased following exposure to IL-1β. Serum levels of free-T3 were measured in PBS (white bars) and IL-1β exposed (grey bars) P5 and P10 mouse pups. Data are shown as mean ± SEM and were obtained with 4–11 samples per group. Asterisks indicate statistically differences obtained by Mann–Whitney test. ^∗^*p* < 0.05; ^∗∗^*p* < 0.01.

In mice exposed to IL-1β from P1 to P5, levels of free-T3 in the blood at P5 were reduced by approximately 20% (Fig. 1). In PBS and IL-1β groups, free-T3 levels increased between P5 and P10. Five days after the cessation of IL-1β exposure at P10 there was no difference in circulating levels of free-T3 (nor free-T4, data not shown) between PBS and IL-1β groups. Analysis of microarray data for genes known to be involved in TH signalling and to be TH-responsive revealed that at P5 and P10 expression of at least 42 genes were moderately but significantly altered by IL-1β exposure (Table 1). KEGG pathway analysis specifically revealed that TH signalling was reduced; of note, *Thra*, *Thrb*, *Trhr*, *Trhr2*, *Smrte*, *Mct8* and *Thada* were decreased at P5, and at P10 expression of *Thrb*, *Trhr2* and *Smrte* was still reduced. Several genes not decreased at P5 displayed decreased expression at P10, including *Thrap3*, *Thrap5*, *Aldh1a1* and *Klf9*. Several genes were also increased at both P5 and P10 in IL-1β exposed mice, including *Icosl*, which is required for thyroid follicular cell proliferation and *Shh*, which decreases T3 availability by actions on *Gli* and *D3*. Expression of TH-related genes was also altered in astrocytes isolated from IL-1β exposed mice at P5 (Supp. Fig. 1).

### 3.2 Treatment with T4 did not recover the inflammation-induced defects in oligodendrocyte maturation or myelinationin neonates or young adults

**Fig. 2:**
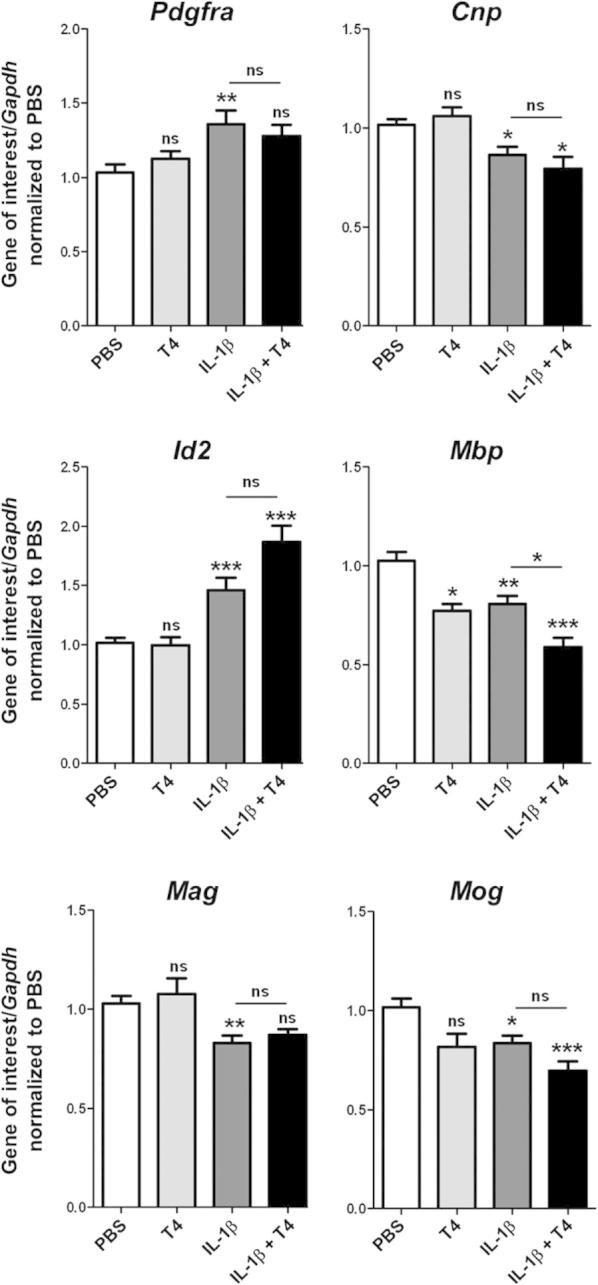
T4 treatment did not prevent IL-1β induced alterations in gene expression of markers of oligodendrocyte maturation and differentiation. Relative gene expression of *Pdgfra*, *Cnp*, *Id2*, *Mbp*, *Mag* and *Mog* were assessed by qRT-PCR from O4-positive cells from P10 mice exposed to PBS (white bars), T4 (light gray bars), IL-1β (dark grey bars) or IL-1β + T4 (black bars). Results are expressed as the mean ± SEM from *n* ⩾ 8 per group. Data were compared two by two (each treatment vs. PBS or IL-1β vs. IL-1β + T4) using the Mann–Whitney test. ^∗^*p* < 0.05; ^∗∗^*p* < 0.01; ^∗∗∗^*p* < 0.001.

In O4-positive cells isolated at P10 from mice exposed to IL-1β from P1 to P5 there was increased expression of genes associated with immature oligodendrocytes (*Cnp*, *Pdgfra*), increased expression of a negative regulator of myelination (*Id2*), and decreased expression of mature oligodendrocyte markers (*Mbp*, *Mag*, *Mog:* Fig. 2), in agreement with data previously reported from whole cortex gene expression in this model. Treatment with T4 did not reduce either these IL-1β-induced defects in oligodendrocyte maturational markers nor expression of myelin genes (Fig. 2). In addition, concurrent treatment of IL-1β-exposed mice with T3 did not recover the IL-1β induced defects in mature and immature oligodendrocyte markers (Supp. Fig. 2).

**Fig. 3:**
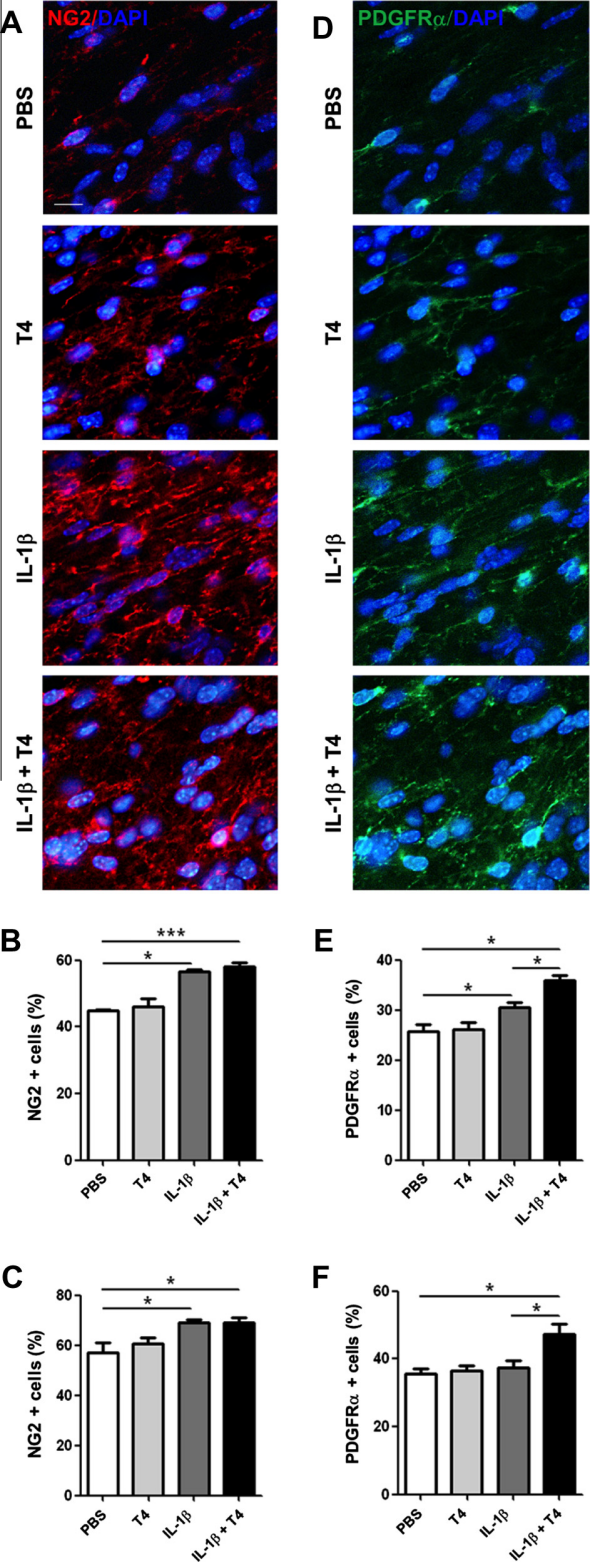
T4 treatment did not prevent the IL-1β induced increase in expression of oligodendrocyte progenitor markers. Expression of oligodendrocyte progenitors markers NG2 (A–C) and PDGFRα (D–F) in the cortical white matter of P5 mice exposed to PBS (white bars), T4 (light gray bars), IL-1β (dark grey bars) or IL-1β + T4 (black bars). NG2 and PDGFRα immunoreactivity in the external capsule (A and D) (scale bar 10 μm). Quantification of NG2 and PDGFRα positive cell number in the external capsule (B and E) and corpus callosum (C and F). Results are expressed as the mean ± SEM from *n* ⩾ 4 per group. Asterisks indicate statistically differences obtained by Mann–Whitney test. ^∗^*p* < 0.05; ^∗∗∗^*p* < 0.001.

**Fig. 4:**
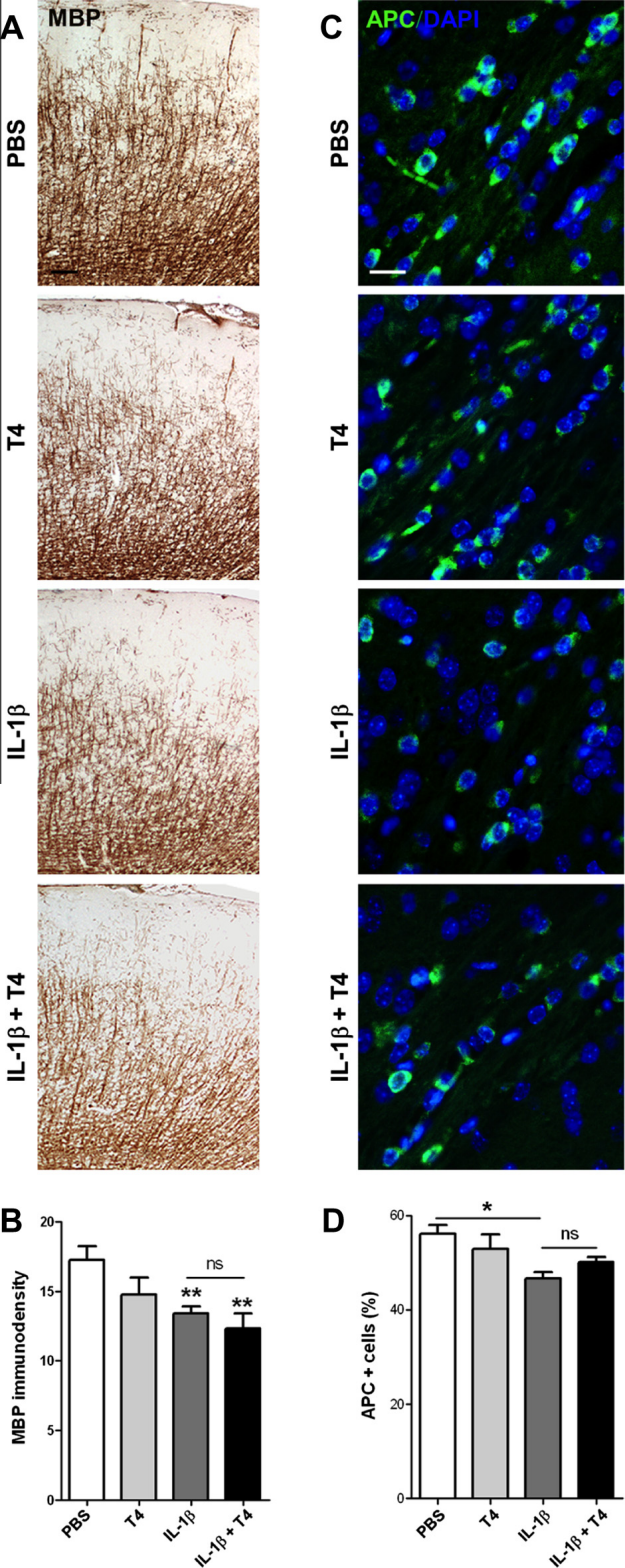
T4 treatment did not prevent the IL-1β induced reduction in myelin or mature oligodendrocyte markers. Expression of mature oligodendrocyte and myelin markers MBP (A and B) and APC (C and D) in P30 mice exposed to PBS (white bars), T4 (light gray bars), IL-1β (dark grey bars) or IL-1β + T4 (black bars). MBP immunoreactivity (A) and quantification (B) within the subcortical white matter (scale bar 100 μm). APC immunoreactivity (C) and quantification (D) in the external capsule (scale bar 20 μm). Results are expressed as the mean ± SEM from *n* ⩾ 4 per group. Asterisks indicate statistically differences obtained by Mann–Whitney test. ^∗^*p* < 0.05; ^∗∗^*p* < 0.01.

The arrest of oligodendrocyte maturation in IL-1β exposed mice was also apparent at P10 using immunohistochemistry for markers of immature oligodendrocytes, NG2 and PDGFRα. As previously reported, we observed an increased density of NG2 and PDGFRα positive cells in the corpus callosum and external capsule (Fig. 3 and Supp. Fig. 3). Treatment with T4 did not reduce the IL-1β-induced increases in the numbers of cells expressing NG2 and further significantly increased the number of cells expressed PDGFRα in the IL-1β + T4 treatment group (Fig. 3 and Supp Fig. 3). There was no change in total numbers of DAPI positive cells in the white matter in any group (data not shown). Treatment with T4 induced a small (≈15%), but significant reduction in free T4 levels at P10 in IL-1β-exposed animals (IL-1β, *n* = 4 52.0 ± 2.1 vs. IL-1β + T4, *n* = 6 43.7 ± 1.8 pmol/l). At P30, immunoreactivity of MBP and numbers of APC positive cells (surrogates for myelination and mature oligodendrocytes, respectively) were reduced by P1–P5 exposure to IL-1β (Fig. 4). T4 treatment did not increase immunoreactive staining for MBP in the sensorimotor cortex, nor APC number in the external capsule compared with animals exposed only to IL-1β (Fig. 4).

## 4 Discussion

We report that in a model of preterm inflammation-induced white matter injury there was no therapeutic effect of TH treatment. Specifically, treatment with T4 (or T3) did not reduce the maturational blockade of oligodendrocytes nor prevent hypomyelination in the neonatal period or in adulthood respectively. These observations are important given the continuing uncertainty over the therapeutic utility of T4 replacement therapy in premature infants (Reuss et al., 1996; Leviton et al., 1999; Simpson et al., 2005) and the relevance of this animal model to the preterm population.

Exposure to systemic perinatal inflammation in this model was designed to mimic the maturational blockade of oligodendrocytes and white matter deficits observed in many premature infants. This is based on the strong clinical associations between prematurity, perinatal maternal/foetal inflammation and white matter damage (Dammann and Leviton, 2004; Wu et al., 2009). Consistent with our previous data, exposure to inflammation induced oligodendrocyte maturational arrest and hypomyelination (Favrais et al., 2011). The current gene expression data is specifically from analysis of purified pre-oligodendrocytes, as opposed to the whole cortex gene expression analysis performed previously. This population-specific data confirms and strengthens the previous association we have reported between inflammation and a blockade of oligodendrocyte maturation in this model.

Immature mice exposed to IL-1β displayed a transient inflammation-induced hypothyroidism. Clinically, hypothyroidism is commonly associated with chorioamnionitis and prematurity (De Felice et al., 2005) and there is a strong correlation between low levels of circulating TH and poor long-term developmental outcome (Simpson et al., 2005; Williams et al., 2005). Hypothyroidism in this model was a strong motivation for trialling T4 as a therapeutic agent, in addition to the numerous observed changes in expression of the TH pathway and TH-responsive genes.

The dosage of T4 we used in this study was higher than the euthyroid dose for preterm infants (8 μg/kg/day over 42 days) (La Gamma et al., 2009) and identical to that used previously, but unsuccessfully, to improve long-term behavioural outcomes in a small clinical trial in preterm infants (Vanhole et al., 1997). The clinically relevant dose of T4 for the neonatal mouse is unknown, however the dose used in this study maintains the normal physiological levels of T4 when administered to adult hypothyroid mice (Rodrigues et al., 2013). Euthyroid treatment is critical as overexposure to TH may have negative effects on the brain (Nicholson and Altman, 1972), and TH repression of TSH can cause hypothyroidism upon completion of therapy (La Gamma et al., 2009). Clinically, treatment with T4 is preferable to T3, as T4 has a considerably higher specific bioavailability within the brain (Forrest et al., 1991). However, as a control for the systemic effects of TH such as those on angiogenesis (Zhang et al., 2010), we also tested T3 in this model and observed no beneficial effect.

In a model of late-preterm HI insult (P7 rat approximating 32–36 weeks GA), no reduction in injury was seen when T4 was administered immediately and at two and four days post-HI at a dose 10-fold greater than used in this study (200 μg/kg) (Hung et al., 2013). This supports our data that T4 may have a limited ability to protect the immature white matter from injury. However, in the HI study a dose of T4 that is 50-fold higher (1000 μg/kg) recovered the loss of myelination, but this indicates a dependency on a supra-clinical dose for neuroprotection (Hung et al., 2013). Furthermore, we have also previously failed to see improvements in neuropathology in an excitotoxicity-induced model of perinatal white matter injury using T3 (Sarkozy et al., 2007). The excitotoxic model is characterized by microcysts but not oligodendrocyte loss (Tahraoui et al., 2001), which, like the IL-1β model is also comparable to injury profiles seen in contemporary cohorts of preterm infants (Billiards et al., 2008; Buser et al., 2012; Verney et al., 2012). Altogether these data suggest that there may be an injury- and age-dependent specificity to neuroprotection with TH. Nevertheless, to comprehensively rule out improvements in brain health due to TH treatment it will also be necessary to assess facets of brain development such as neuronal maturation and neurite outgrowth/synaptogenesis. Cognitive and behavioral testing may be required to visualize improvements such as these in the gray matter and to measure any improvements not identified from structural assessments of the white matter.

We should also consider that an extended treatment regime might be necessary to reveal beneficial effects of TH, and whether the timing of TH treatment in this study mimics in any way that applied to infants. Clinically, TH is administered to infants pre-exposed to inflammation and for 42 days after birth, spanning the period of maturation of pre-oligodendrocytes into immature-oligodendrocytes (Back et al., 2001). This period of maturation is approximately P2–P7 in the mouse (Craig et al., 2003), and we administered TH from P1 to P5, during a great proportion of this maturational process. Regarding the timing of TH treatment, the time course of maternal/fetal inflammation precipitating premature birth is poorly understood (Hagberg et al., 2012). However, inflammation discernable from plasma cytokine analyses persists for at least seven days in preterm infants born with funisitis or chorioamnionitis, and is suggested to be even longer lasting (Leviton et al., 2011). As such, the inflammatory process may be well established before initiation of treatment in many cases of preterm birth. However, any therapy will be required to show efficacy during an ongoing inflammatory process, suggesting that the timing of TH treatment in this model is of some clinical relevance. Indeed, administering TH during inflammation in this model may mean that inflammation-induced decreases in TH signalling might have been substantial enough to abrogate any beneficial effects of T4. However, although many genes within the TH signaling pathway are dysregulated by inflammation in this model, the specific fold changes are moderate (median, −1.2) suggesting that this pathway is likely to be impaired, but not completely unresponsive to supplementation. Nevertheless, delayed treatment, and/or combination therapy with anti-inflammatory drugs may be required to reveal the true neuroprotective potential of TH treatment.

In conclusion, this study suggests that further work is needed to understand the role of TH in protection/repair across the currently available animal models of perinatal injury, and in particular with a focus on the effects of inflammation. Importantly, given the paucity of experimental research knowledge applicable to the preterm population, this study also suggests that careful consideration should be made before additional clinical trials exploring the therapeutic efficacy of TH are initiated. Following a longitudinal study that suggested improved outcome for early preterm infants(van Wassenaer et al., 1997), a large double blind randomised clinical trial was begun in 2012 powered for *a priori* testing of infant outcome stratified by gestational age at birth (Ng et al., 2013). This new trial with its full assessment of infant outcomes by gestational age is critical for our understanding of the potential of TH therapy, as in the original 1997 trial a subgroup of children (those born at 29 weeks) actually had worse behavioural outcomes following TH treatment (van Wassenaer et al., 2002). Thus caution should be exercised in assuming that at worst any trial using TH therapy will find limited efficacy. We suggest that any potential for TH treatment to reduce the immense burden of neurological impairment caused by perinatal brain injury may be unlocked by understanding its role in the context of insult type and severity.

## Conflict of interest

Nothing to report.

## Authorship and contributorship

A.L.S., J.V.S. and D.C. performed the animal experiments and MACS, qRT-PCR, hormone measurements and immunohistochemistry. H.H. and M.A. performed the gene expression analysis. A.L.S., J.V.S., D.C., H.H., P.G. and B.F. participated in experimental design, interpretation of data and preparation of the manuscript.

## Figures and Tables

**Table 1:** Systemic inflammation induces dysfunction of TH related genes and pathways in oligodendrocytes of the immature brain. Following microarray gene expression analysis the processed signal intensity values were compared using *t*-test (*p* ⩽ 0.05) with a Benjamini-Hochberg multiple testing correction. The gene ID, statistically significantly fold change (red = down-regulation; green = up-regulation), details of gene function and role in white matter injury where applicable are presented.



