# Supplementary material for: Failure of thyroid hormone treatment to prevent inflammation-induced white matter injury in the immature brain
Source: Brain Behav Immun. 2014 Mar;37(100):95–102. doi: 10.1016/j.bbi.2013.11.005 (PMC3969588; doi:10.1016/j.bbi.2013.11.005)
Supplement: Supplementary data 1 — Supplementary figures and table. [file mmc1.doc]

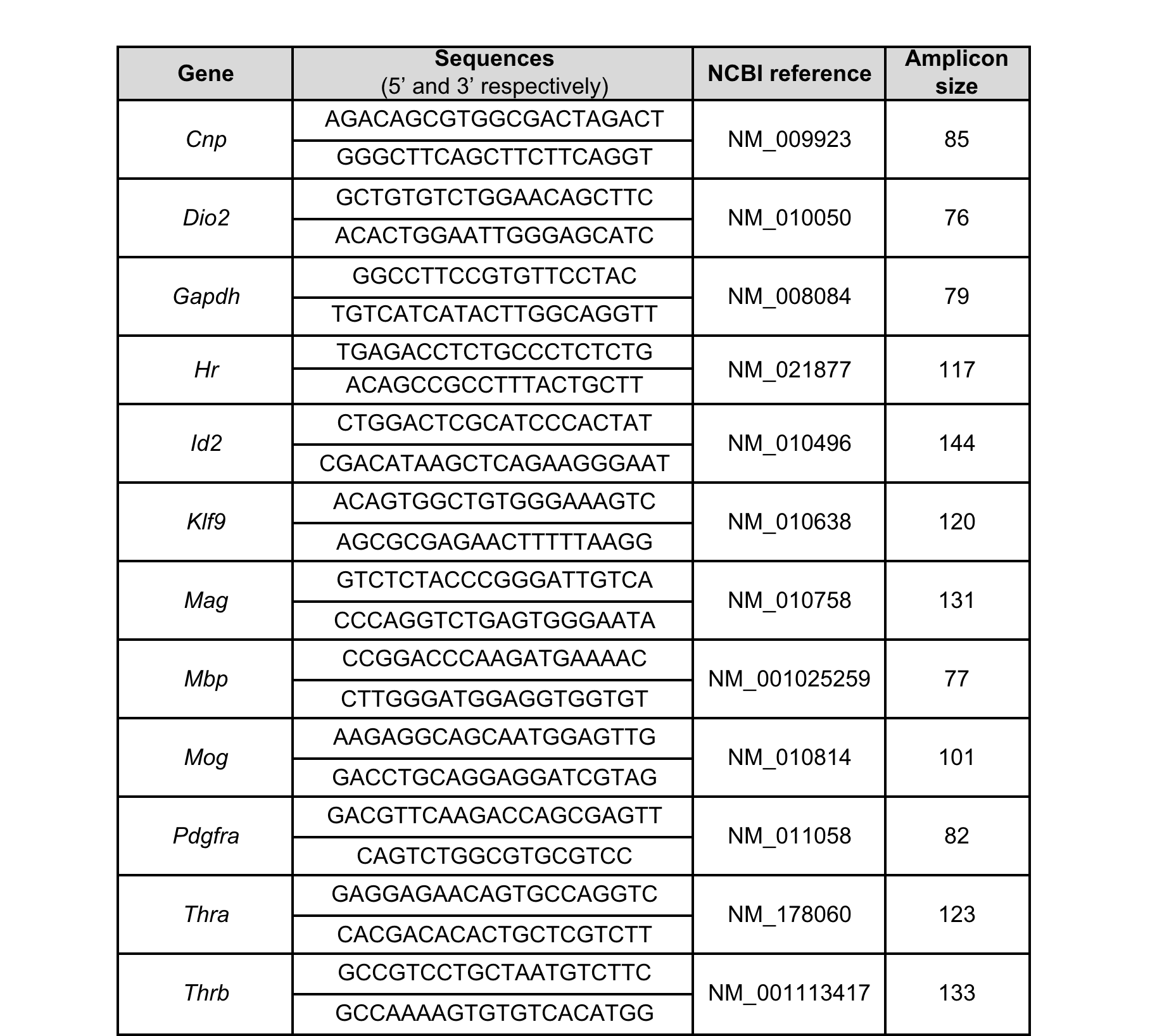


**SUPPLEMENTARY TABLE 1: Primer sequences and NCBI reference for primers for qRT-PCR**

**
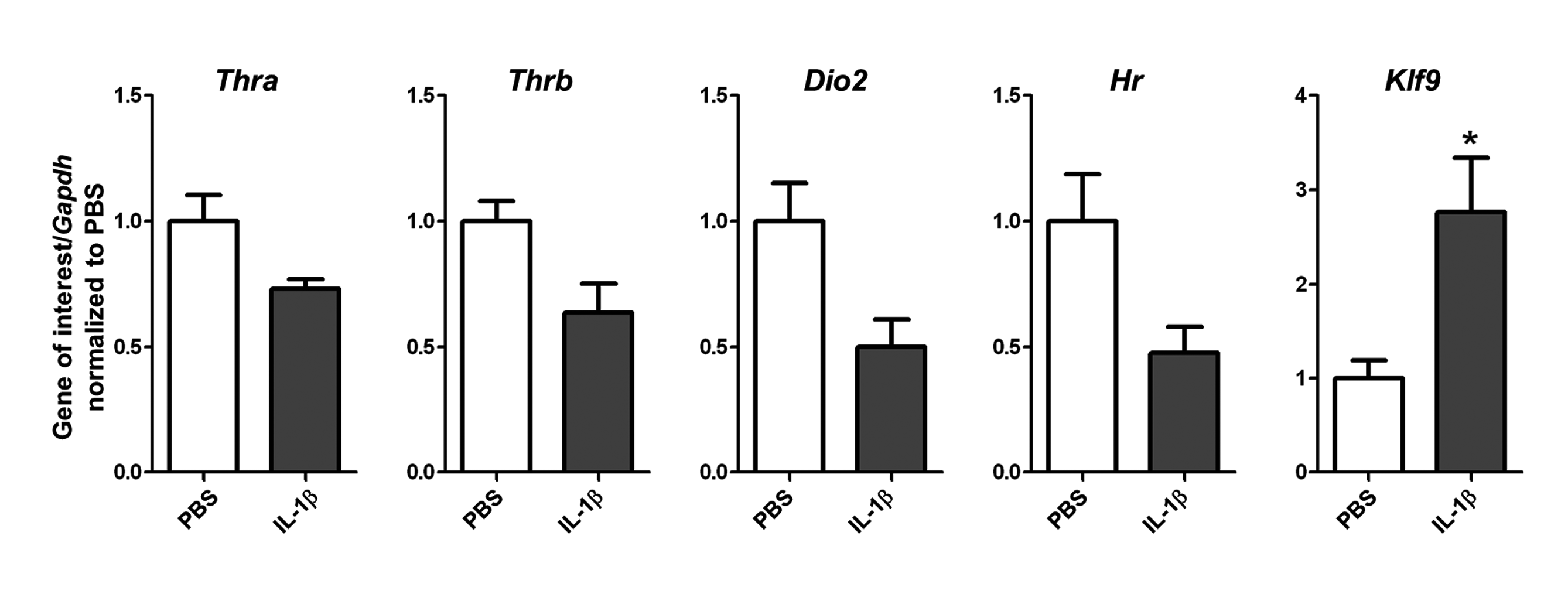
**

**SUPPLEMENTARY FIGURE 1:** **Expression of TH related genes was deregulated in astrocytes isolated by MACS from PBS (white bars) or IL-1β (grey bars) exposed mice at P5.**  Astrocytes were extracted using magnetic beads coupled to the GLAST-antibody (Miltenyi BioTec) and qRT-PCR performed. Results are expressed as the mean±SEM from n=4 animals per group. Asterisks indicate statistically differences obtained by Mann-Whitney test. *p<0.05.


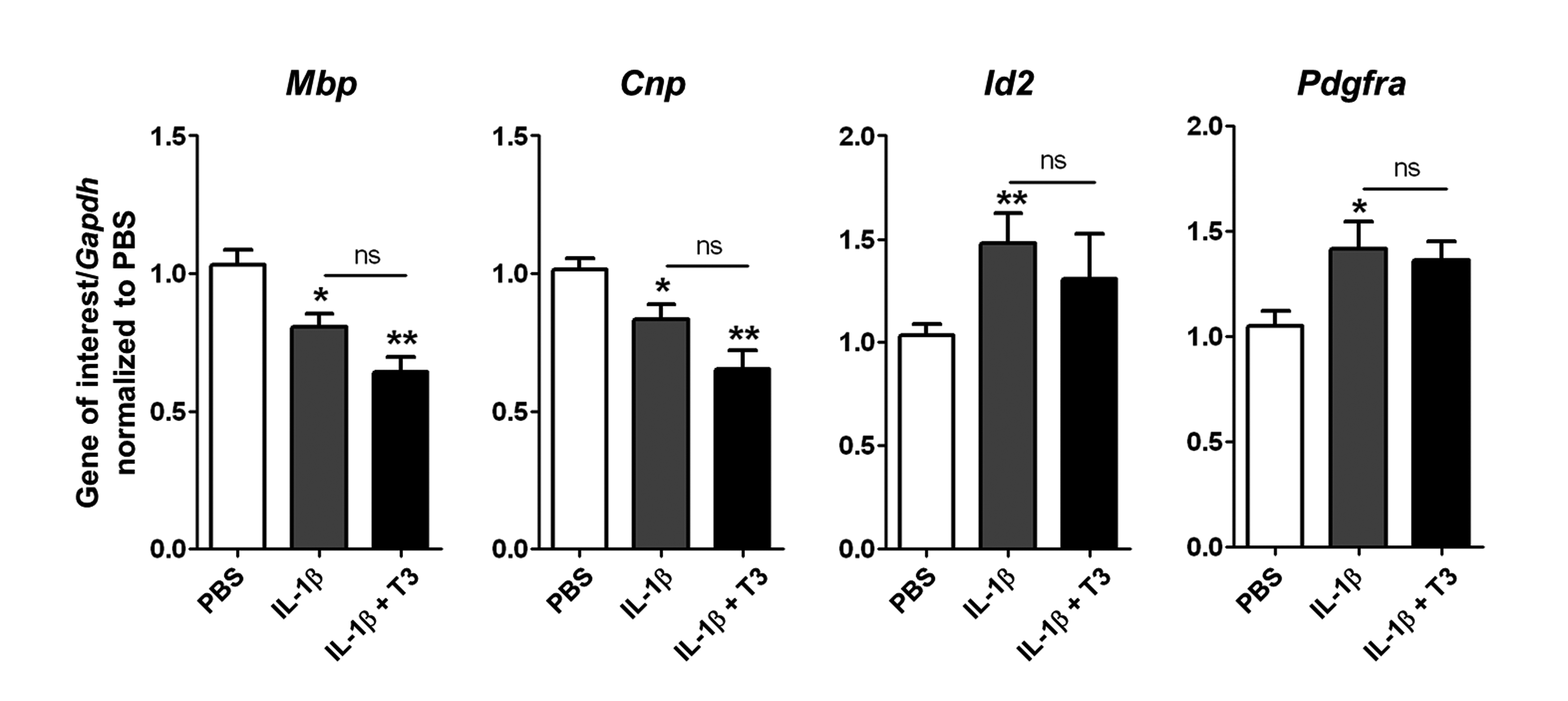


**SUPPLEMENTARY FIGURE 2:** **T3 treatment did not prevent IL-1β induced alterations in gene expression of markers of oligodendrocyte maturation and differentiation.** Relative gene expression of *Mbp, Cnp, Id2 and Pdgfra* were assessed by qRT-PCR from O4-positive cells from P10 mice exposed to PBS (white bars), IL-1β (dark grey bars) or IL-1β+T3 (black bars). Results are expressed as the mean±SEM from n≥5 per group. Data were compared to PBS or IL-1β using the Mann-Whitney test. *p<0.05; **p<0.01.


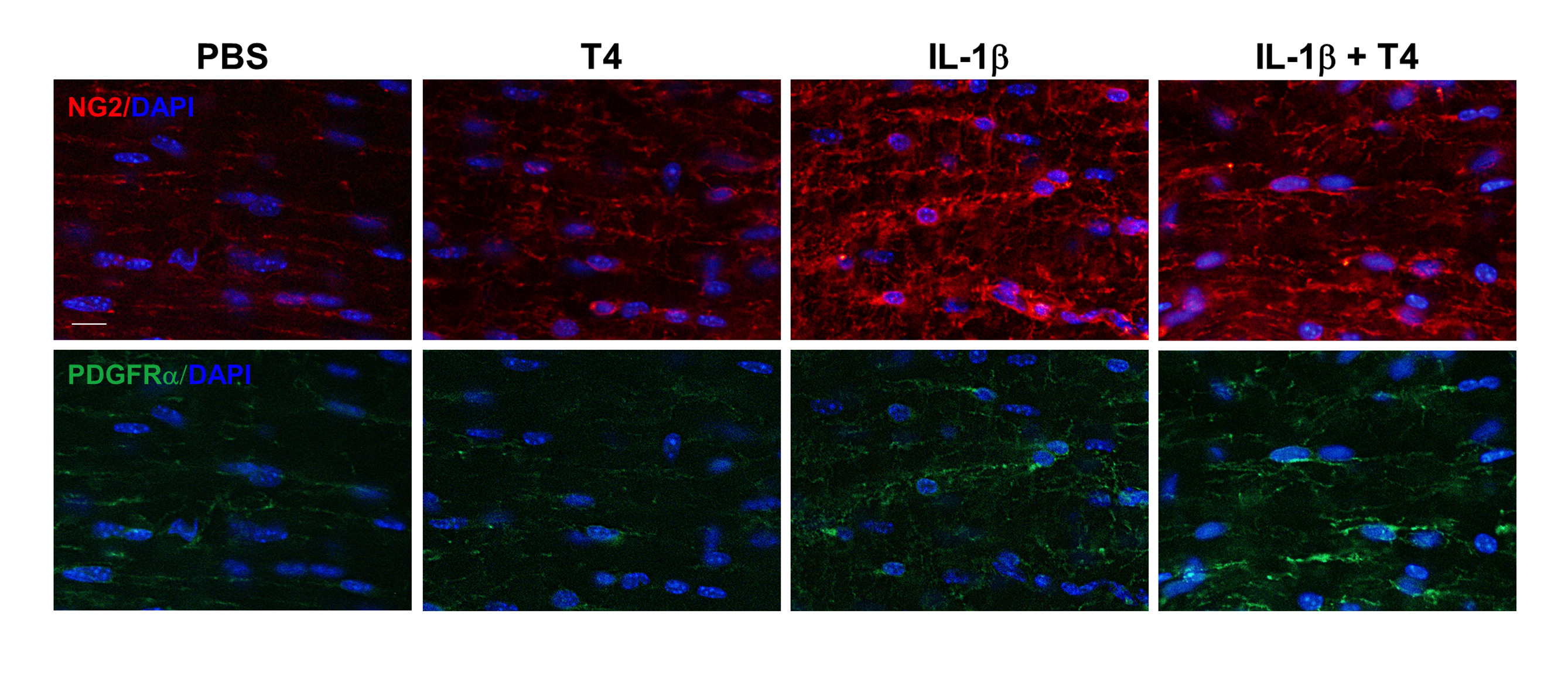
**SUPPLEMENTARY FIGURE 3: T4 treatment did not prevent the IL-1β induced increase in expression of oligodendrocyte progenitor markers in the corpus callosum.** Immunohistological localization in frozen 16µm thick fixed/frozen sections of oligodendrocyte progenitors markers NG2 and PDGFRα in the corpus callosum of P5 mice exposed to PBS, T4, IL-1β or IL-1β+T4. Scale bar 10µm
